# Characterization of Adenomatous Polyposis Coli Protein Dynamics and Localization at the Centrosome

**DOI:** 10.3390/cancers8050047

**Published:** 2016-04-30

**Authors:** Christina Lui, Myth T. S. Mok, Beric R. Henderson

**Affiliations:** 1 Centre for Cancer Research, The Westmead Institute for Medical Research, The University of Sydney, Westmead, NSW 2145, Australia; christina.lui@sydney.edu.au (C.L.); mythmok@mythmok.com (M.T.S.M.); 2 School of Biomedical Sciences, The Chinese University of Hong Kong, Hong Kong SAR, China; 3 Shenzhen Research Institute, The Chinese University of Hong Kong, Shenzhen, China

**Keywords:** APC, centrosome, colorectal cancer, FRAP, protein dynamics, Wnt signaling

## Abstract

The adenomatous polyposis coli (APC) tumor suppressor is a multifunctional regulator of Wnt signaling and acts as a mobile scaffold at different cellular sites. APC was recently found to stimulate microtubule (MT) growth at the interphase centrosome; however, little is known about its dynamics and localization at this site. To address this, we analysed APC dynamics in fixed and live cells by fluorescence microscopy. In detergent-extracted cells, we discovered that APC was only weakly retained at the centrosome during interphase suggesting a rapid rate of exchange. This was confirmed in living cells by fluorescence recovery after photobleaching (FRAP), which identified two pools of green fluorescent protein (GFP)-APC: a major rapidly exchanging pool (~86%) and minor retained pool (~14%). The dynamic exchange rate of APC was unaffected by C-terminal truncations implicating a targeting role for the N-terminus. Indeed, we mapped centrosome localization to N-terminal armadillo repeat (ARM) domain amino acids 334–625. Interestingly, the rate of APC movement to the centrosome was stimulated by intact MTs, and APC dynamics slowed when MTs were disrupted by nocodazole treatment or knockdown of γ-tubulin. Thus, the rate of APC recycling at the centrosome is enhanced by MT growth, suggesting a positive feedback to stimulate its role in MT growth.

## 1. Introduction

Adenomatous polyposis coli (APC) is a multifunctional tumor suppressor protein and its mutation contributes to the progression of colorectal cancer (CRC) [1,2,3]. APC is a key regulator of the oncogenic protein β-catenin in the Wnt signaling pathway. In response to ligand-activation of Wnt at the plasma membrane, the β-catenin destruction complex containing APC no long assembles, enabling β-catenin to accumulate in the nucleus. This leads to the activation of a network of genes that promote cell immortalization, proliferation and migration [4,5,6]. The cell-protective properties of APC are particularly well characterized in the hereditary polyp syndrome known as familial adenomatous polyposis (FAP). Clinical studies showed that patients with specific APC gene mutations correlated with higher numbers of polyp manifestations and were predisposed to increased risk of colorectal malignancies. The most common mutation occurs in the “mutation cluster region”, in particular at amino acid 1309 in APC and additional truncating mutations within the second allele greatly increase disease progression to CRC [7,8,9]. 

In addition to maintaining β-catenin concentrations, APC is also a regulator of microtubules (MTs) and APC mutations influence MT integrity and growth [10,11]. In mitosis, cells expressing a truncated mutant form of APC tend to show a weakening of MT-chromosome attachments and exhibit irregular mitotic spindle morphologies that lead to chromosome separation errors, followed by genetic instability [12,13,14,15], a typical characteristic of cancer [16].

Many anti-cancer drugs target dividing or mitotic cells to control cell proliferation in tumors; however, there is an increasing interest in targeting interphase cells, especially with a focus on the MT system [17,18]. In interphase, APC localizes to the cell edge at membrane protrusions that drive cell motility in migration [19,20]. APC is an MT plus-end protein that stabilizes MT polymerization either via direct binding through its basic domain or indirectly via binding to the MT regulating protein, EB1 [10]. MTs originate from the centrosome, also known as the “MT organizing center” (MTOC). We recently confirmed that cells expressing the truncated mutant APC1-1337 display a reduced rate of MT regrowth at the centrosome compared to full-length APC in cancer cell lines [21]. Moreover, in that study, the loss of full-length APC slowed the rate of early MT growth (nucleation). Thus, given that the interphase APC-assisted growth and stabilization of MTs is linked to normal cell features such as membrane protrusion and directed cell migration, which are disrupted by APC mutations in cancer cells [10], part of the tumor suppressive role of APC is likely to involve the stimulation of MT growth at centrosomes in both interphase and mitosis [22,23]. 

While it is known that both mutant and full-length forms of APC locate at centrosomes, there are major gaps in our understanding of the targeting, dynamics and regulation of APC at this structure. To address this, we describe here the mapping of the minimal APC centrosome-targeting sequence for the first time, characterized in live cells the dynamic exchange rate of APC at the centrosome. We demonstrate a modest but unexpected positive-feedback regulation wherein MT growth stimulates the on-rate of APC at the centrosome, providing a mechanism to amplify the impact of APC on MT growth. 

## 2. Results

### 2.1. Full-Length and Truncated APC Localize to the Centrosome Throughout the Cell Cycle and Display High Dynamic Exchange during Interphase

**Figure 1:**
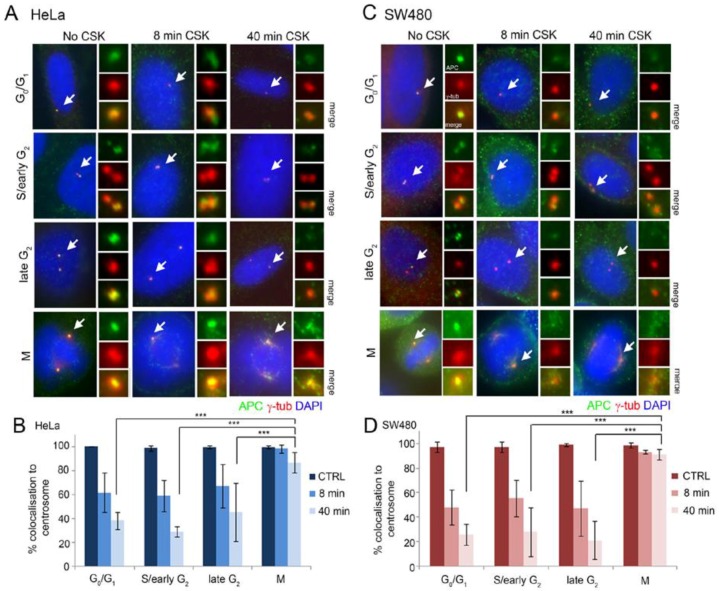
APC is less retained at centrosomes in interphase than mitotic cells. Immunofluorescence micrographs of APC at centrosomes after CSK detergent buffer washout in (**A**) HeLa cells (express full-length APC (APC-FL)), and (**C**) SW480 (express mutant APC1-1337). Cells were stained with antibodies against APC (Ab7 mAb, **green**) and γ-tubulin (**red**) to detect centrosomes (**white arrows**). The localization of retained APC was scored at different stages of the cell cycle, which were classified according to centrosome number and position (G_o_/G_1_ = 1 centrosome, S/G_2_ = 2 centrosomes, late G_2_ = separated centrosomes that have not yet matured, M = divided centrosome with increased γ-tubulin at the centrosome). APC localization was visually scored by microscopy after 8 min and 40 min detergent washout, and compared to cells with no detergent treatment (No CSK) (*n* = 300). (**B** and **D**) Scores pooled from three experiments were normalized to indicate the percentage of centrosome localization based on ability to clearly visualize APC by direct fluorescence microscopy with adjustment of the *Z*-axis focus (mean ± SD) (***, *p* < 0.001). Additional cell images of APC in mitotic cells are shown in Figure S1.

Centrosome functions vary with each phase of the cell cycle, coinciding with recruitment of specialized proteins. To study APC localization at the centrosome during the cell cycle, centrosomes were marked with γ-tubulin antibody and cells were co-stained for endogenous APC, analysing APC-FL (full-length) in HeLa cells and APC1-1337 (mutant) in SW480 cells (Figure 1). APC staining was compared at four approximate cell cycle stages (G_0_/G_1_, early S/G_2_, late G_2_ and mitosis), which were classified based on centrosome number and morphology as performed in previous studies [22,24] (see Figure 1 legend for details). Immunofluorescence microscopy revealed strong APC staining at the centrosome at all stages of the cell cycle in 90%–100% of endogenous untreated cells (see “No CSK” cell images in Figure 1A,C). Similar results were observed for both APC-FL and APC1-1337 mutant with different antibodies (specificity of staining patterns confirmed recently by siRNA [21,25]) indicating that localization to the centrosome does not change throughout the cell cycle when APC is truncated. 

Next, we applied an *in vitro* detergent extraction assay [26,27] to compare the degree of APC retention at centrosomes during the cell cycle. CSK buffer (see methods) was used to remove soluble/mobile proteins from cells and enable detection of the insoluble/immobile proteins that are usually anchored to cellular structures. HeLa and SW480 cells were treated with CSK buffer for 8 min or 40 min, followed by fixation with methanol:acetone and staining for APC. The degree of APC retained at the centrosome was visually scored by microscopy. This was a binary measurement (yes or no staining detected) based on individual scoring. The retention pattern of APC-FL in HeLa cells was very similar to that of the mutant APC1-1337 in SW480 cells, and both forms of APC were retained in only a fraction of cells (20%–40% of total) in G_1_, S or G_2_-phase (Figure 1B,D). In contrast, both forms of APC were resistant to detergent extraction and retained at centrosomes (and to some extent in centrosome-proximal structures, see Figure 1A,C and Figure S1) in mitosis, with >90% of all mitotic cells scored exhibiting APC anchorage after CSK treatment. This is suggestive of a change in function and in protein complex formation of APC at the centrosome in preparation for mitosis, and that these properties are at least partly conserved in CRC since the APC mutant was also retained at the mitotic centrosome and some proximal (possibly MT-associated) sites. The data indicate that, in interphase cells, APC is likely to undergo dynamic exchange at the centrosome. 

### 2.2. Wild-Type and Cancer Mutant APC Display Similar Dynamics at the Centrosome: Identification of Mobile and Immobile Pools of APC

**Figure 2:**
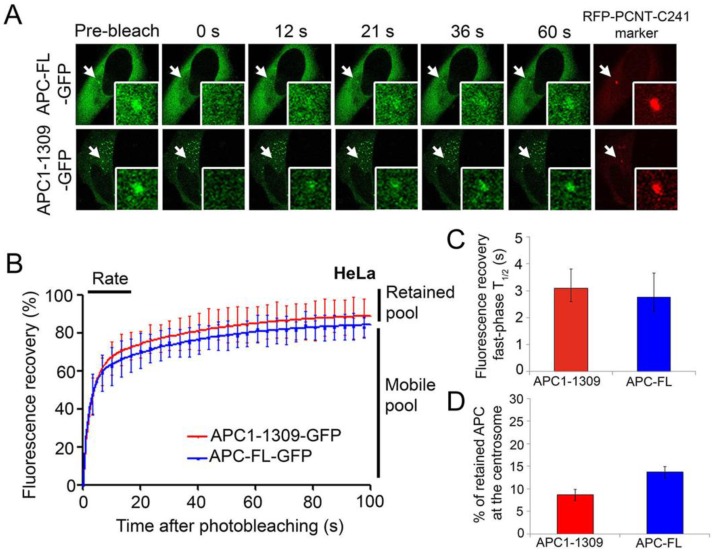
APC-FL and APC1-1309 display similar fast dynamics at the centrosome in live cells. (**A**) pAPC-FL-GFP and pAPC1-1309-GFP (GFP = green fluorescent protein, **green**) were independently co-transfected with pRFP-PCNT-C241 (**red**) into HeLa cells for 48 h. We first determined that the minimal bleaching time required to completely diminish the centrosome pool of GFP-tagged APC was 3 s with 100% laser intensity in fixed cells. FRAP was performed on the expressed fluorescent proteins by live cell confocal microscopy using the laser for photobleaching of centrosome fluorescence, followed by time-lapse image capture (60× magnification). The baseline fluorescence was acquired by taking a pre-bleached image, followed by sequential imaging at 0 s, 12 s, 21 s, 36 s and 60 s post-bleach. The insets show higher magnification views of the centrosome. (**B**) Fluorescence recovery curves are shown of APC at the centrosome, indicating relative rates of recovery and equilibration (plateau) at the centrosome for up to 100 s after bleaching. (**C**) The fast-phase half time in seconds (T_1/2_ mean ± SD) and the (**D**) retained fraction (% mean ± SD; extrapolated from curves that had not quite reached plateau) are shown for each protein from a pooled average of 15–20 cells based on Graph Pad Prism analysis. The dynamic profiles of APC-FL and APC1-1309 were not significantly different.

In order to measure APC dynamics at the centrosome, we used the fluorescence recovery after photobleaching (FRAP) technique to track APC-GFP on/off-rates in living cells. In the FRAP assay, GFP-tagged proteins are expressed in cells then subjected to laser-induced photobleaching at the centrosome, following which recovery of the fluorescent protein is measured accurately using confocal microscopy with image acquisition at specific time intervals (see methods). This allows for the quantification of protein exchange dynamics, in particular on-rates and degree of retention *in vivo*. In the case of centrosomes which are non-membranous, the dynamics measured after bleaching represent a direct exchange between centrosome and the cytoplasm [28]. Analysed in HeLa cells, GFP-tagged APC constructs were co-expressed with a red fluorescent protein (RFP)-tagged C-terminal fragment of pericentrin (pRFP-PCNT-C241) to mark the centrosome (Figure 2A). The FRAP assay data was analysed in GraphPad Prism 5 (GraphPad Software Inc., La Jolla, CA, USA) to determine the rate of fluorescence recovery over time, and the relative size of the dynamic and immobile pools of APC (Figure 2B–D).

We discovered that GFP-tagged APC-FL and APC1-1309 share a similar dynamic profile at the centrosome (Figure 2). In each case, the fluorescence recovery curves revealed two quite distinct pools of APC at the centrosome, a mobile pool and a retained pool. The rapid fluorescence recovery observed within the first 100 s represents the more dominant mobile pool, which reflected 86.2% of total centrosomal APC-FL and 91.3% of centrosomal APC1-1309 (Figure 2B and Table 1). The mobile pools exhibited a rapid recovery that could be further divided into two distinct phases; the fast phase kinetics of APC (measured in the first 40 s) had a low T_1/2_ of ~3 s (Figure 2C), while the slow phase T_1/2_ was ~26–27 s (Table 1). It is the “fast” phase pool that displayed the greatest recovery of APC at the centrosome and is most reliably quantified using GraphPad Prism software (see values and levels of significance in Table 1). In addition to the dynamic pool, a small fraction of APC was retained or immobile at the centrosome during the period of analysis, and this is indicated by the difference between maximal recovery plateau and the pre-bleach value on the recovery curves. The predicted retained pools measured here in living cells comprised 13.8% and 8.7% of the total centrosomal pools of full-length and truncated mutant APC, respectively (Figure 2D and Table 1). This is within a similar “minority” range as the retained APC pools observed after *in vitro* detergent extraction (Figure 1). Overall, these recovery rates shown for APC are comparable to those reported for other centrosomal proteins such as aurora A and nek2A kinases with T_1/2_ values of 2.1 and 3.2 s, respectively [29,30]. The current study is the first to specifically determine APC dynamics at the centrosome. 

### 2.3. Intact Microtubules Stimulate Transport of APC to the Interphase Centrosome 

**Figure 3:**
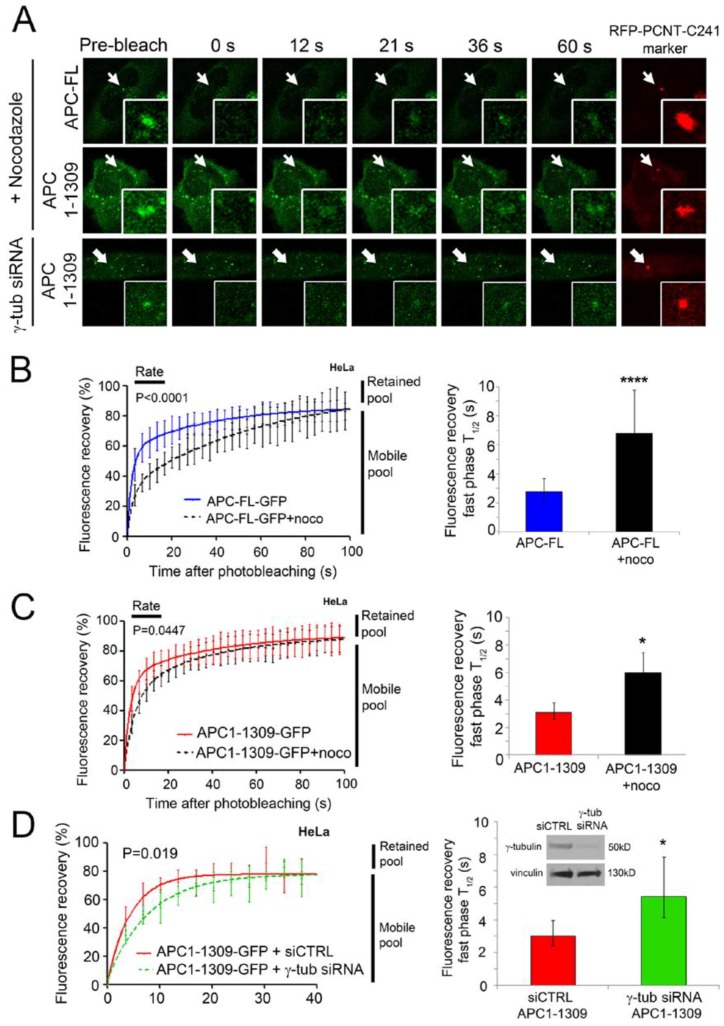
APC full-length and mutant dynamics at the centrosome are slowed by nocodazole treatment. (**A**) pAPC-FL-GFP and pAPC1-1309-GFP (**green**) were each co-transfected with pRFP-PCNT-C241 (red) into HeLa cells. APC-GFP was analysed for dynamic recruitment at the centrosome by FRAP in the presence and absence of 33 µM nocodazole. The effect of γ-tubulin on APC dynamics was also tested where FRAP was performed after depletion with γ-tubulin siRNA. (**B**) Fluorescence recovery curves were plotted as shown for APC-FL, indicating relative rates of recovery and equilibration (plateau) at the centrosome for up to 100 s after bleaching. The presence of nocodazole (**black dashed line**) significantly reduced the rate of recovery of APC-FL-GFP compared to that of untreated cells (**blue line**) (*n* = 20–30). This was also indicated by comparison of T_1/2_ values for the fast recovery pools (T = 0–40 s) (*p* < 0.0001), and extrapolated retention levels, calculated from the recovery curve data using Graph Pad Prism software as above (see Table 1). (**C**) The dynamic exchange profile of APC1-1309 at the centrosome +/− nocodazole (**black dotted line**) showed a small difference in the dynamic rate of recruitment compared to untreated cells (*p* = 0.0447). There was a small but significant difference in T_1/2_ value; however, no change in retention after nocodazole treatment. (**D**) Fluorescence recovery curves are shown for APC1-1309 for siCTRL (**red**) and γ-tubulin siRNA (**green**) transfected cells (*n* = 9–10). Confirmation of γ-tubulin knockdown was by Western blot, and vinculin was used as loading control. Column graph shows the T_1/2_ of the fluorescence recovery over 40 s, which was significantly increased after the knockdown of γ-tubulin (*p* = 0.0194). No significant change in the maximum recovery (retention) was detected. (*, *p* < 0.05; ****, *p* < 0.0001).

APC is well known to bind and stabilize microtubules [31,32], and, therefore, we tested whether the intact MT network affected APC dynamics at the centrosome. In early experiments, we found that, as previously reported [22], disruption of MTs by nocodazole did not prevent the steady-state APC localization at the centrosome in fixed cells. However, FRAP assays identified a surprising influence of MTs on APC dynamics. In living cells, FRAP was performed on APC-FL-GFP expressed in HeLa cells that were untreated or treated with 33 µM nocodazole for 1 h prior to the commencement of the assay. The experiment revealed that fluorescence recovery of APC-FL-GFP was significantly slowed at the centrosome after disruption of MTs by nocodazole treatment (Figure 3A,B). The fast-phase T_1/2_ (*i.e*., time required to reach half-maximal recovery rate) was increased significantly by three-fold from ~2 s to ~7 s (Figure 3B, right panel). Not only did nocodazole cause a delay in the rate at which ectopic APC-FL moved toward the centrosome, but computer-based analysis of the recovery curves suggested it might have modestly altered the level of retained APC predicted from the 100 s post-bleach time period measured (Table 1). Therefore, loss of MT coherence elicits a marked delay in the rate of APC-FL movement to the centrosome. 

**Figure 4:**
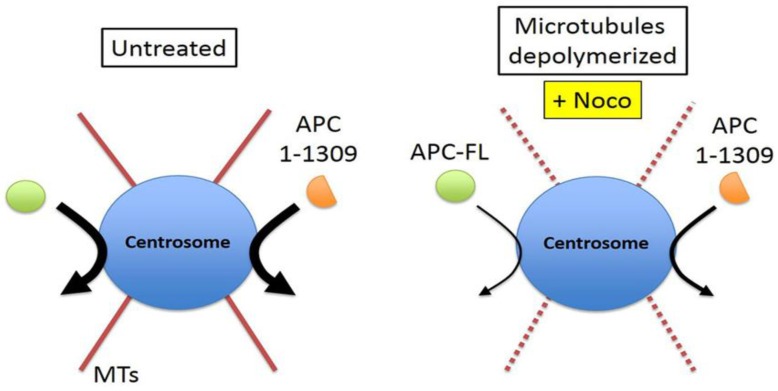
Model to summarize the effect of microtubules on APC dynamics at centrosome. In the physiological state APC-FL and APC1-1309 show similar dynamics with a high exchange rate (**left**) at the centrosome, however this rate is reduced by the depolymerization of MTs and the depletion of γ-tubulin (**right**).

The effect of nocodazole was also tested by FRAP on APC1-1309-GFP at the centrosome (Figure 3A,C). As shown, nocodazole likewise slowed the rate of recovery of the ectopic mutant APC, as indicated by a higher T_1/2_ (Figure 3C, right panel), although it was not quite as pronounced as the delay measured for APC-FL. Notably, centrosome retention of the GFP-tagged APC1-1309 was unchanged in live cells after nocodazole treatment (Table 1). In summary, while drug induced MT depolymerization did not affect the overall localization of APC at the centrosomes, it did slow the rate of APC movement to the centrosome and this was observed for both mutant and full-length protein. This suggests that MTs assist in transport of APC from the cytoplasm to centrosome during the rapid exchange phase of association (see model outlined in Figure 4).

### 2.4. γ-Tubulin Stimulates the Rate of Recruitment of APC to the Centrosome 

γ-tubulin is an essential component of the γ-tubulin-ring complex (γ-tuRC), which is important in the nucleation of MTs [33,34]. We showed above that MTs contribute to the efficient transport of APC to the centrosome (Figure 3A–C). To test if APC movement to the centrosome is affected by γ-tubulin (the strongest MT nucleator), FRAP analysis was performed in control cells and cells depleted of γ-tubulin. HeLa cells were treated with control siRNA (siCTRL) or γ-tubulin siRNA for 24 h, followed by the co-expression of GFP-tagged APC1-1309 with RFP-tagged PCNT-C241 (to mark the centrosome) for another 48 h (Figure 3A, lower panel). The knockdown efficiency was confirmed by Western blot analysis (Figure 3D inset, right panel). APC1-1309 was analysed because it expresses more consistently in transient assays than APC-FL. Fluorescence recovery curves revealed that the siRNA-mediated loss of γ-tubulin caused a modest but significant reduction in the recovery rate of APC1-1309 to the centrosome during the first 40 s post-bleaching (*p* = 0.019) (Figure 3D). This was reflected in the measured T_1/2_ where γ-tubulin depletion increased the T_1/2_ by 100%, from ~3 s to ~6 s (Figure 3D and Table 1). The retention of APC1-1309 was not significantly affected by γ-tubulin loss, with maximal fluorescence recovery at ~83%–87% in both control and γ-tubulin siRNA transfected cells (Table 1). The ~12%–16% retention of APC observed was consistent with the previous FRAP experiment of Figure 2. Since the FRAP data for γ-tubulin knockdown corresponds closely to that seen after depolymerization of MTs, we propose that the loss of γ-tubulin indirectly affects APC by reducing MT formation (Figure 4). As a specificity control we note that siRNA-mediated depletion of another MT-regulating core centrosome protein, pericentrin (PCNT), did not alter APC1-1309 on-rate dynamics (Figure S2).

### 2.5. The Centrosome Targeting Sequence of APC Maps to the N-terminal ARM Domain Sequence 334-625

**Figure 5:**
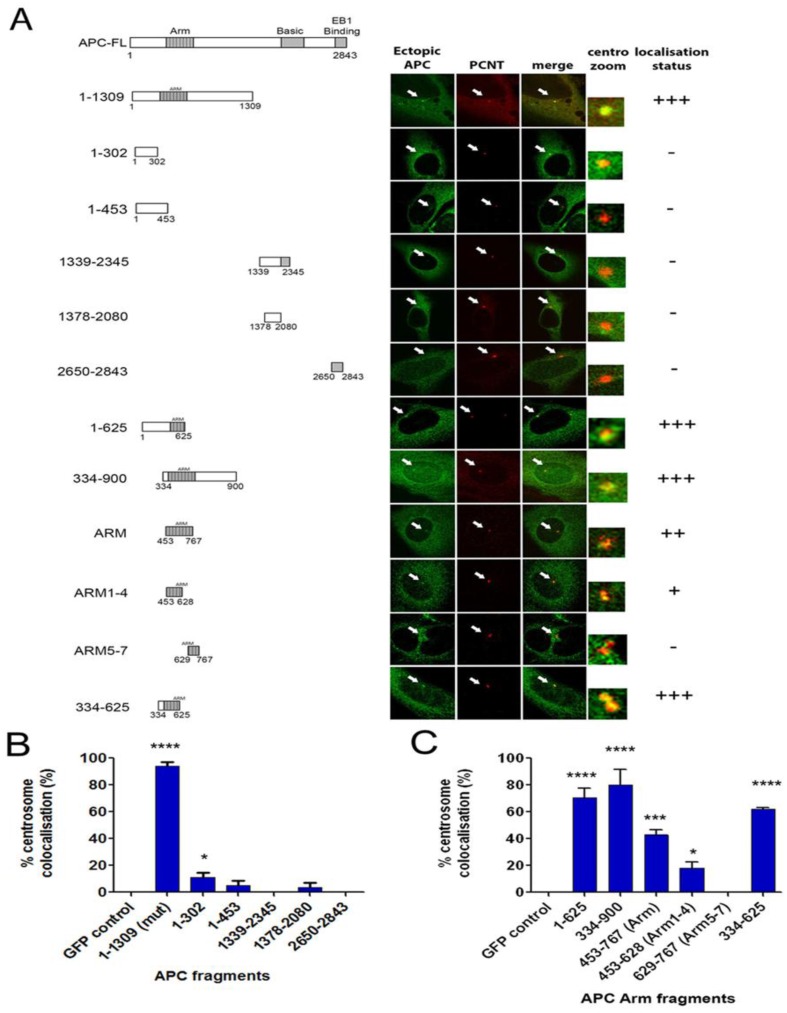
The armadillo repeat (ARM) domain efficiently targets APC to the centrosome. (**A**) schematic diagram of the APC protein and various fragments tested for centrosome localization. All fragments were GFP-tagged (**green**) and overexpressed in U2OS cells followed by a co-staining of PCNT (**red**) to mark the centrosome. Optical sections were acquired with an Olympus confocal FV1000 microscope (Olympus, Tokyo, Japan) and subsequent images were used to score co-localization. Representative images (cell and centrosome zoom) are shown for each expressed construct. (**B**) Scoring was based on the % of transfected cells with APC detected at the centrosome. Data was collected from three independent experiments, scoring at least 100 cells per experiment (mean ± SD). A students *t*-test analysis was used to determine which GFP-APC fragments were located at the centrosome at a significantly higher level than GFP alone (*, *p* < 0.05, **, *p* < 0.01, ***, *p* < 0.001, ****, *p* < 0.0001). The minimal centrosome targeting sequence mapped was 334–625 overlapping the ARM domain.

To define the centrosome targeting sequence, we performed deletion mapping on APC (Figure 5). A series of GFP-tagged APC peptide fragments were transiently expressed in U2OS cells and compared for staining at the centrosome (marked by co-staining for PCNT). Optical cross-sections were acquired by confocal microscopy and images were analysed and scored for centrosome co-localization. APC1-1309-GFP was our main positive control and localized strongly and consistently to the centrosome in >90% of cells as expected, and this high level of co-staining is indicated by the score “+++” (Figure 5A). The C-terminal APC fragments 1379–2080, 1339–2345 and 2650–2843 revealed no significant centrosomal co-localization (“-“). This was consistent with an earlier study where the N-terminal APC fragment 1–750 amino acids localized to the centrosome [14]. However, a detailed mapping revealed that certain N-terminal fragments that located upstream of the ARM domain, *i.e*., 1–302 and 1–453 (Figure S3), did not localize at the centrosome (Figure 5B). A large fragment, 334–900, that encompasses the ARM domain and flanking sequence displayed a very strong staining at centrosomes (+++), as did the N-terminal sequence 1–625 which comprises the first half of the ARM repeat domain (Figure 5C and Figure S3). Thus, a series of ARM-containing sequences was compared, mapping the core targeting sequence to 334–625, which comprises both N-terminal flanking sequence and ARM repeats 1–4. While we cannot exclude that some centrosome targeting may be mediated by an extended C-terminal sequence, our data show that the 334–625 sequence recruited GFP to the centrosome in 60% of cells, and its N-terminal location explains why truncated cancer mutant forms of APC are often detected at the centrosome.

## 3. Discussion

This study has shown that APC is highly dynamic at the centrosome suggesting a series of transient interactions and the potential for rapid regulation of its centrosomal concentration. Regulatory proteins frequently display a dynamic flux at the centrosome as revealed by FRAP studies of GFP-tagged proteins in living cells [30,35,36]. To determine the kinetics of APC-GFP pools at the centrosome, we applied FRAP and identified two quite distinct APC populations in living cells. The major pool of APC turns over very rapidly (T_1/2_ ~ 3 s), leaving only a minor fraction (~14% of the total) that resides stably at the centrosome (Figure 2). A similar result was obtained using an *in vitro* detergent-based assay (Figure 1). This dynamicity of APC at the centrosome is in stark contrast to a previous FRAP analysis where the majority of APC at membrane protrusions/tips was found to be anchored quite stably [37]. This indicates that most of the centrosome-localized APC is recruited through weak or transient protein-protein interactions, and its rapid exchange may serve a regulatory function or act to signal changes in the cytoplasmic microenvironment to the centrosome. 

The dynamic profile of APC at the centrosome was surprisingly unaffected by loss of the C-terminal half (~1534 amino acids) of the protein (Figure 2), indicating that all the key sequences that mediate transient and strong protein associations reside within the N-terminal region of APC. Thus, truncating cancer mutations affect the functionality [21] but not the targeting/dynamics of APC at the centrosome. In the context of comparing APC with other key Wnt signaling regulators, we note that the dynamic profile was somewhat similar to the APC binding partner and major Wnt nuclear transactivator protein β-catenin, which, in FRAP experiments reported by others, was found to recover up to 95% after photobleaching of the centrosome [38]. In contrast, APC dynamics differed markedly to the APC partner B56α, a subunit of protein phosphatase 2A, which was well retained at the interphase centrosome [39]. We observed profound differences in the *in vitro* anchorage of APC at the centrosome during the cell cycle, with the APC population shifting from highly mobile to strongly retained as cells entered mitosis (Figure 1). This is somewhat reminiscent of γ-tubulin which, in FRAP assays, changed from 50% immobile to almost exclusively anchored at the centrosome as cells progressed from interphase to mitosis [36,40]. In both scenarios, a larger proportion of γ-tubulin, an integral protein of the centrosome [41,42,43,44], was anchored at the centrosome and may be available to help recruit APC.

APC binds directly to MTs through its “basic” domain [45], and indirectly through kinesin motor complexes contacting its armadillo domain [46]. The latter finding revealed MT plus end directed movement of APC. Here, we suggest the possibility that APC may move along MTs in a minus-end directed manner toward the centrosome. In FRAP assays, we showed that depolymerization of the MT network by nocodazole caused a significant delay in the on-rate of APC-GFP at the centrosome, leading to a two-fold increase in T_1/2_ (Figure 3B). The disruption of MTs slowed the centrosome recruitment rate of both full-length and mutant APC (Figure 3B,C), narrowing the responsive region mainly to the N-terminal half of APC. Recently, we confirmed that APC associates with γ-tubulin via the N-terminal amino acids 1–453 of APC [21]. This could implicate γ-tubulin in the MT-regulated recruitment of APC (FL and mutant) to the centrosome. Here, we showed that silencing of γ-tubulin caused a slowing of APC transport to the centrosome similar to that seen with nocodazole. This suggests that weak or transient interactions between APC and γ-tubulin may contribute to the on-rate of the mobile pool of APC. Alternatively, it is possible that loss of γ-tubulin indirectly affected recruitment of APC through disruption of MT nucleation and assembly (Figure 3). This latter scenario seems more likely given that deletion mapping pinpointed the minimal centrosome localization sequence of APC to a region (334–625) that lies adjacent to the γ-tubulin binding site (Figure 5). We propose that, while MTs are not necessary to deliver APC to the centrosome, the presence of an intact MT network “stimulates” this recruitment. Whether this involves MT minus-end directed motors (e.g., dynein) or “capture” mechanisms that associate with the 334–625 sequence remains to be determined. At this stage, we cannot exclude that MT-tip capture of APC may also be compromised by nocodazole treatment and influence its presence at the centrosome.

In summary, our data implicate APC as a mobile scaffold [47], with its N-terminus directing it to the centrosome and mediating specific weak interactions with integral centrosome proteins (e.g., γ-tubulin), while its C-terminus is predicted to assemble additional protein complexes required to regulate MT nucleation [21] and other activities. It will be interesting to define in future the role of interacting proteins, and of self-association mediated by domains near the Arm repeats [48,49], in APC targeting and function at the centrosome. 

## 4. Materials and Methods

### 4.1. Cell Lines and Drug Treatment

Human HeLa cervical carcinoma cells and U2OS osteosarcoma cells were obtained from The Westmead Institute Centre for Cancer Research cell line repository and confirmed mycoplasma negative. SW480 CRC cells were purchased from the commercial cell repository CellBank Australia. Cells were cultured in Dulbecco’s modified Eagle’s medium (DMEM) supplemented with 12% fetal bovine serum, 2% penicillin/streptomycin, 4-(2-hydroxyethyl)-1-piperazineethanesulfonic acid (HEPES) and glutamine and cultured at 37 °C in a humidified chamber in the presence of 5% CO_2_. To achieve MT depolymerization, nocodazole (Sigma) was used to treat cells using 33 µM of nocodazole for 1 h at 37 °C under conditions optimized as recently described [21]. 

### 4.2. Plasmids and Transfection

pAPC-FL-GFP was a generous gift from Dr. Angela Barth (Stanford University), APC fragments of 334–625 and 1–453 were cloned from pCMV-APC (obtained from Prof. Bert Vogelstein, Johns Hopkins University, Baltimore, MD, USA) into the pEGFP-N1 vector (Clontech, Mountain View, CA, USA), using Sal1 and BamH1 sites. The 334–625 amino acid sequence was amplified by PCR using forward (5’-GA TCG AGG TCG ACG ATG CTA GCT ATG TCT AGC TCC CAA GAC-3’) and reverse (5’-TAC TTA TGG ATC CCG CTG GCT CCG GTA AGT AAG AGT GC-3’) primers. The APC 1–453 sequence was PCR amplified with forward (5’-GAT CGA CGT CGA TGG CTG CAG CTT CAT ATG ATC AG-3’) and reverse (5’-TAC TTA TGG ATC CCG TAG AAC ACA CAC AGC AGG ACA G-3’) primers. pCMV-APC (full-length), pAPC1-1309-GFP and pAPC1379-2080-GFP were prepared as described previously [19]. DNA (2 µg–3 µg) was transfected into cells with either Fugene HD reagent (Promega, Madison, WI, USA), Lipofectamine 2000 reagent (Invitrogen, Thermo Fisher Scientific, Waltham, MA, USA) or polyethylenimine (PEI; Polysciences Inc., Warrington, PA, USA) according to manufacturer instructions. Control siRNA and pericentrin-B (PCNT-B) targeting siRNA (Qiagen S104190781) and γ-tubulin siRNA (Santa Cruz sc-29323) were transfected into cells using Lipofectamine 2000 (Invitrogen, Thermo Fisher Scientific, Waltham, MA, USA). At 4–6 h post-transfection, the media mix was removed and replaced with fresh media as described above. Cell harvest occurred 48–72 h post-transfection. 

### 4.3. Centrosome Targeting Sequence Mapping Using Immunofluorescence Staining and Microscopy

U2OS cells were plated onto coverslips in 6-well plates and grown until 60%–70% confluency before transfection of GFP-tagged APC fragment sequences for 48 h (see Section 4.2). Cells were fixed with methanol:acetone (1:1) for 3 min, followed by three washes using PBS. Cells were blocked with 3% bovine serum albumin and stained with primary antibodies. To detect the centrosome, polyclonal PCNT (Abcam ab4448, 1:1500) and PCNT monoclonal (Abcam ab28144, 1:1500) were used. γ-tubulin (Sigma T5192, 1:800) and monoclonal γ-tubulin (Sigma T5326, 1:800) antibodies have also been used to yield a similar localization result. Cells were washed three times using PBS and subsequently incubated with the fluorescence secondary probes Alexafluor-594 (Invitrogen, Thermo Fisher Scientific, Waltham, MA, USA). Cells were washed extensively before they were mounted with Vectashield (Vector Laboratories Inc., Burlingame, CA, USA). Cells were stained in the dark to prevent bleaching of GFP. Cells were visualized using Olympus BX-51 inverted microscope (Olympus) and optical sections were taken using an Olympus FV1000 confocal microscope (Olympus) at 60× magnification. 

### 4.4. Cell Retention Assay 

To compare centrosome retention, a detergent extraction assay was used to remove soluble proteins from HeLa and SW480 cells, respectively. Cells were grown on poly-L-lysine (Sigma, 0.1 mg/mL) coated coverslips and then incubated in CSK extraction buffer (10 mM Pipes, pH6.8; 300 mM sucrose; 5 mM MgCl_2_; 100 mM NaCl; 0.5% Triton X-100) for 0, 8 and 40 min at 32 °C. Cells were gently washed with PBS and then fixed using methanol:acetone. Immunofluorescence staining was performed as above. Ab7 monoclonal (or H290 polyclonal) was used to detect APC and γ-tubulin antibody to detect centrosomes. Cells were scored for centrosome localization using Olympus BX-51 fluorescence microscope at 40× magnification (Olympus). Images were taken using a SPOT RT slider camera 23.1 (SPOT Imaging, Sterling Heights, MI, USA) connected to the microscope. Data was collected from three independent experiments with over 100 cells per treatment condition. γ-tubulin was used to define stages of the cell cycle based on the appearance of centrosomes. 

### 4.5. Fluorescence Recovery after Photobleaching (FRAP) 

pAPC-FL-GFP and pAPC1-1309-GFP were co-transfected with pRFP-Pericentrin C241 in HeLa cells for 48–72 h. Transfection of siRNA was performed 24 h after the transfection of GFP expression plasmids. For FRAP assays with nocodozole, 33 µM of nocodazole was added 1 h prior to the assay and cells were incubated at 37 °C. The assay was performed in a humidified CO_2_ chamber at 37 °C and cells were analysed using an Olympus FV1000 confocal laser-scanning microscope with 60× (Olympus) water objective. The RFP-pericentrin was imaged as a marker to locate the centrosome. Then, pre-bleached images were taken twice (laser power 15%–20%), the region of interest was photo-bleached for 2–3 s with 100% 488 nm laser power intensity and post-bleach imaging continued for up to 120 s at ~1.2 s intervals. For nocodazole treated FRAP assays, cells were pre-treated with 33 µM of nocodazole for 1 h at 37 °C before FRAP analysis. Settings such as laser power, zoom, scan speed and duration of bleach were kept constant throughout each experiment. Data analysis on the series of images was performed with Olympus FluoView Version 1.6a software (Olympus). The average intensities in all regions of interest including the centrosome, background signal and the whole cell fluorescence bleaching were calculated and exported to Microsoft Excel using the program. The final fluorescence intensity at each recovery time-point was calculated after accounting for photo-bleaching caused by imaging and deduction of background fluorescence. Final data were entered into GraphPad Prism 5 and determined to best fit a two-phase association curve. T_1/2_ and plateau values were determined and used to compare initial speed of recovery and the recovery percentage at the centrosome. The fraction of protein that contributes to the recovery is called the “mobile” fraction and the protein that does not is called the “immobile” fraction. Each type of FRAP analysis was based on 15–20 cells from at least three independent experiments. 

## 5. Conclusions

This study reveals that APC is not static at the interphase centrosome but displays a dynamic exchange. A range of experimental approaches showed that the full-length and cancer-truncated forms of APC displayed very similar kinetics of movement at the centrosome, consistent with the major targeting sequence being localized to the N-terminal half of the protein. We further showed that the rapid on-rate of APC at the centrosome was at least partly enhanced by intact MTs. APC was recently shown to stimulate growth of MTs at the interphase centrosome [21], and thus our data suggest a potential feedback regulation to ensure optimal accumulation of APC at the centrosome. 

## Figures and Tables

**Table 1:** FRAP ^1^ Summary of T_1/2_ and retention of APC-FL and APC1-1309 at the centrosome.

APC +/− Treatments	Mobile Pool ^2^ (%)	Retained Pool ^2^ (%)	T_1/2_ Fast Pool ^3^ (s)	T_1/2_ Slow Pool (s)
APC-FL	86.23	13.77	2.77	25.58
APC-FL+ noco	97.30	2.70	6.79 (*p* < 0.0001)	42.67
APC1-1309	91.31	8.69	3.09	27.82
APC1-1309+noco	90.39	9.61	5.99 (*p* = 0.045)	25.79
APC1309 +control siRNA	87.81	12.19	3.02	23.34
APC1309 + γ-tub siRNA	83.77	16.23	5.44 (*p* = 0.019)	10.52

^1^ The analysis of fluorescence recovery of GFP-tagged APC-FL and APC1-1309 at the centrosome in HeLa cells (see Figure 2 and Figure 3) was measured using a combination of Excel and algorithms from the GraphPad Prism 5 program. Given the period of recovery measured (100 s), we could only reliably measure levels of significance for differences in the fast phase pool (1–40 s), our major focus in this study. The Table shows values for centrosome retention and recovery rates of fast and slow populations with and without treatment. All fluorescence recovery curves were fitted to a two-phase recovery curve producing two T_1/2_ values (fast and slow). See Methods for further details. ^2^ Mobile/retained pools: plateaus reached on the curves were used to determine (or predict in mostcases since recovery curves had not always reached plateau within the time period of analysis) the distribution of mobile and retained pools measured by the % of maximum fluorescence recovery by the Graph Pad Prism 5 program. ^3^ Rate of recovery: The Table indicates the speed of recovery of fluorescence for the two populations of APC at the centrosome. The fast pool T_1/2_ values were based on the recovery speed during the first 40 s (T = 0–40 s). The slow pool data shown is an extrapolation and included only for reference.

## Supplementary Materials

The following are available online at http://www.mdpi.com/2072-6694/8/5/47/s1, Figure S1: Additional images of APC staining in mitotic cells after 40 min CSK extraction, Figure S2: Knockdown of pericentrin does not affect APC1-1309 dynamics at the centrosome in live cells, Figure S3: Confirmation of newly cloned GFP-tagged APC constructs.

## Abbreviations

The following abbreviations are used in this manuscript:

APC: adenomatous polyposis coli
ARM: armadillo repeat
CRC: colorectal cancer
CSK: cytoskeletal extraction buffer
FRAP: fluorescence recovery after photobleaching
GFP: green fluorescent protein
RFP: red fluorescent protein
γ-tuRC: γ-tubulin ring complex
MT: microtubule
MTOC: microtubule organizing center
PCNT: pericentrin
