# Supplementary material for: Characterization of Adenomatous Polyposis Coli Protein Dynamics and Localization at the Centrosome
Source: Cancers (Basel). 2016 Apr 30;8(5):47. doi: 10.3390/cancers8050047 (PMC4880864; doi:10.3390/cancers8050047)
Supplement: Supplementary file 1 [file cancers-08-00047-s001.pdf]

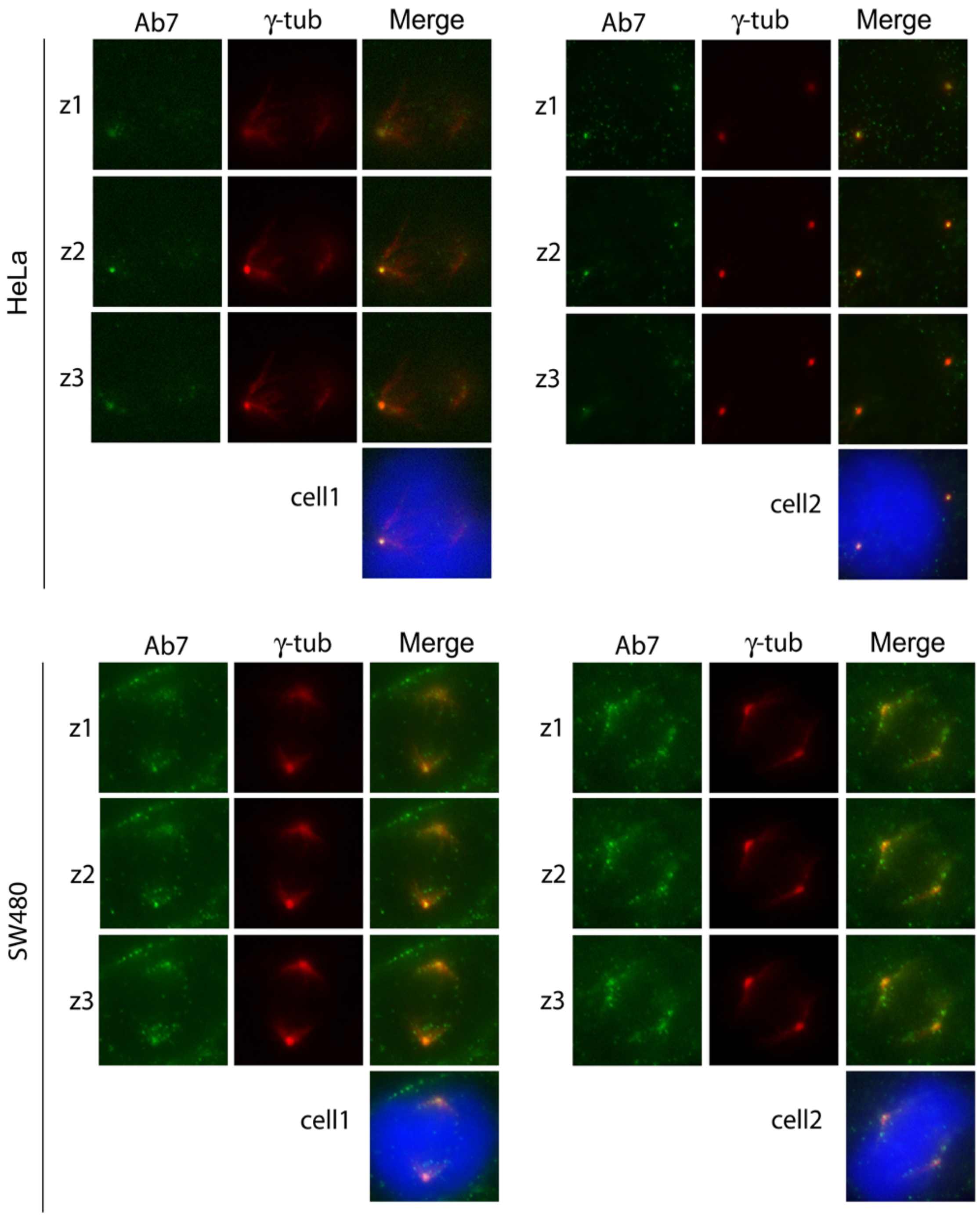

Figure S1. Z-stack images of mitotic cells stained for APC after detergent extraction. Mitotic HeLa cells and SW480 cells were stained for APC (Ab7 - green) and counterstained for γ-tubulin (red) following 40 min CSK extraction as described in Methods and shown in Figure 1. These additional z-axis viewpoints show detection of APC at the centrosome but also confirm in SW480 cells some centrosome-proximal staining at spindle-associated sites.

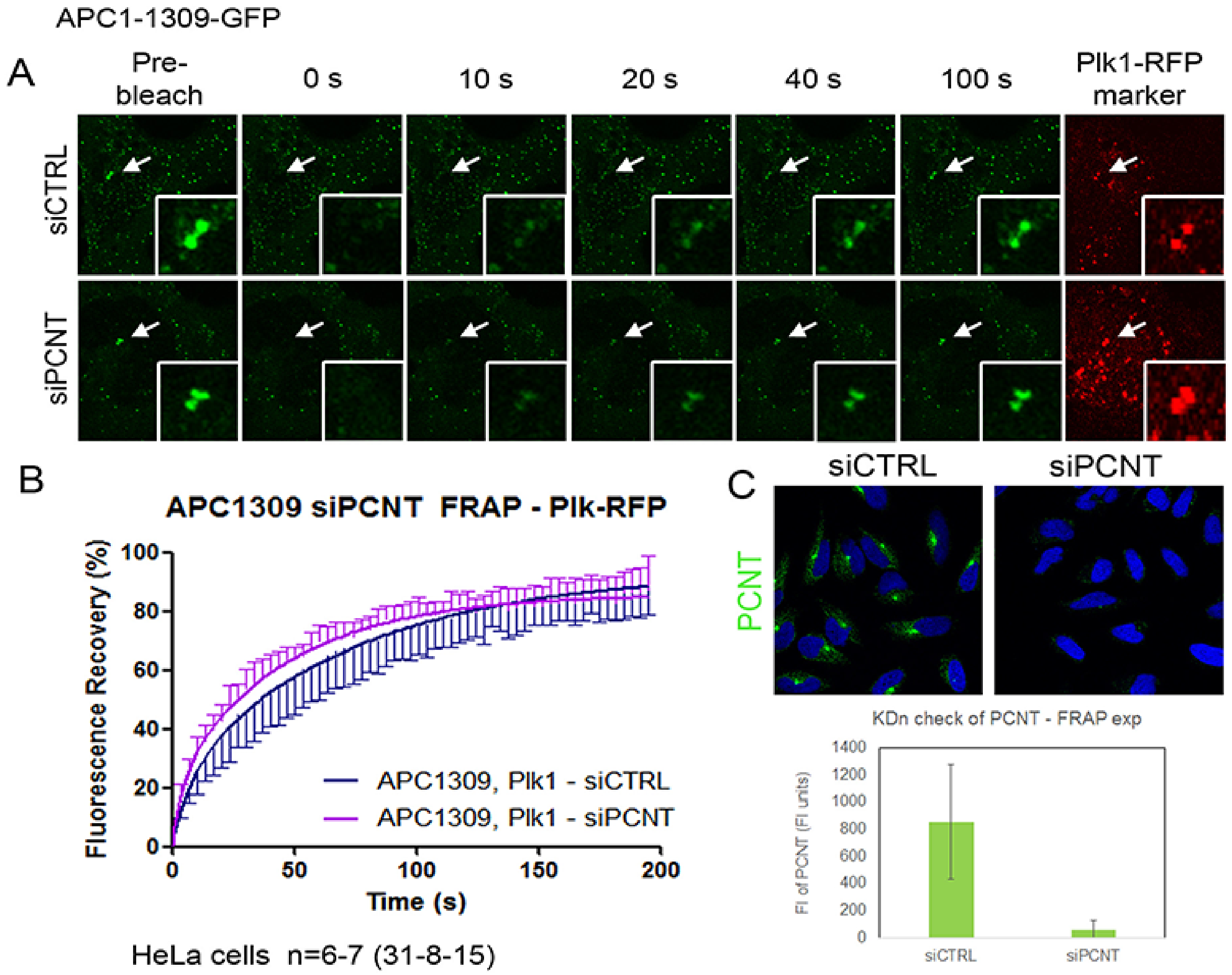

Figure S2. Knockdown of pericentrin does not affect APC1-1309 dynamics at the centrosome in live cells. (A) HeLa cells were treated with either siCTRL or PCNT-B siRNA, followed by the co-transfection of pAPC1-1309-GFP (green) with pPlk1-RFP centrosome marker (red) for 48 h. FRAP was performed on the expressed fluorescent proteins by live cell confocal microscopy using the laser for photobleaching of centrosome fluorescence, followed by time-lapse image capture (60X magnification). The baseline fluorescence was acquired by taking a pre-bleached image, followed by sequential imaging post-bleach. The insets show higher magnification views of the centrosome. (B) Fluorescence recovery curves are shown of APC at the centrosome, indicating relative rates of recovery and equilibration (plateau) at the centrosome for up to 200 s after bleaching. The recovery curves represent the average of 6-7 cells analysed with GraphPad Prism software. The dynamic profiles of APC treated with siCTRL or PCNT-B siRNA were not significantly different. (C) Knockdown of PCNT was efficient as shown.

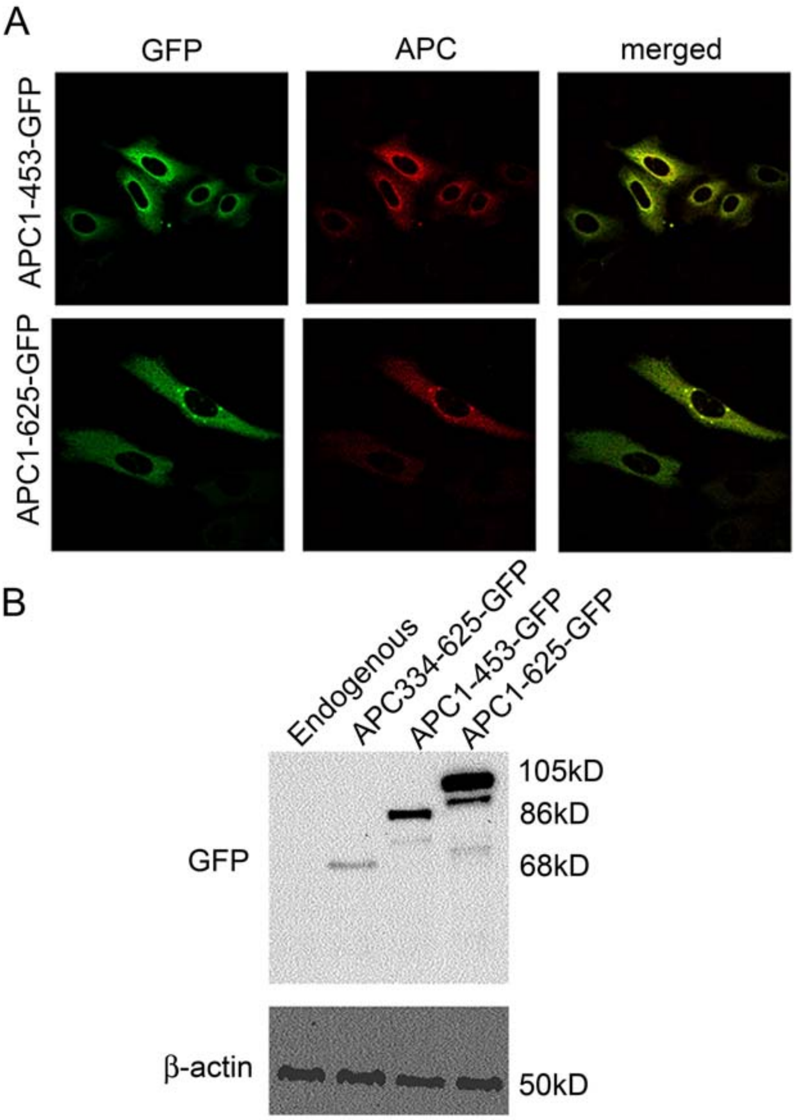

Figure S3. Confirmation of newly cloned GFP-tagged APC constructs. The sequences APC334-625, APC1-453 and APC1-625 were excised from a pCMV-APC construct and inserted into a pEGFP vector. (A) Newly cloned GFP-tagged APC1-453 and APC1-625 were overexpressed (green) in HeLa cells and their expression validated by staining with Ab7 mAb (red) (that detects the N-terminus of APC). (B) All cloned fragments were ectopically expressed in HeLa cells and cells were lysed using RIPA lysis buffer. 50 μg the total lysate was used for Western blot to confirm the correct size of the new clones. GFP-tagged proteins were detected using GFP rabbit antibody. The sizes shown indicated the correct sizes for each clone. β-actin was detected as loading control.
